# Supplementary material for: Forest edges increase pollinator network robustness to extinction with declining area
Source: Nat Ecol Evol. 2023 Jan 30;7(3):393–404. doi: 10.1038/s41559-022-01973-y (PMC9998274; doi:10.1038/s41559-022-01973-y)
Supplement: Supplementary file 1 — Supplementary Methods 1–5, Results 1 and 2, and Tables 1–5. [file 41559_2022_1973_MOESM1_ESM.pdf]

nature ecology & evolution

Article https://doi.org/10.1038/s41559-022-01973-y

Forest edges increase pollinator network robustness to extinction with declining area

In the format provided by the authors and unedited

Supplementary Materials for

Forest edges increase pollinator network robustness to extinction with declining area

Peng Ren, Raphael K. Didham, Mark V. Murphy, Di Zeng, Xingfeng Si, Ping Ding

Table of Contents:

Supplementary Methods 1: 2

Flower resource availability 2

Estimating flower abundance and size 2

Supplementary Methods 2: 3

Sample completeness 3

Supplementary Methods 3: 4

Species composition 4

Supplementary Methods 4: 5

Causal hypotheses for the structural equation model 5

Supplementary Methods 5: 8

Null model approach 8

Null model I: Constraining to the same number of transects 9

Null model II: Constraining both network abundance and network size 9

Supplementary Results 1: 15

Species composition at edge and interior of islands 15

Supplementary Results 2: 15

Sampling results at edge and interior of islands 15

Table S1. Site attributes for the mainland and 41 island sites, with mean plant and pollinator community attributes for transects at edge and interior locations over 3 years sampling combined 20

Table S2. List of plant species observed and their cumulative floral resource availability (m^2) after 20 surveys in 3 years 21

Table S3. List of pollinators observed and their cumulative abundance at all sites over 3 years 23

Table S4. Summary of partial regression coefficients for the final piecewise SEM model describing the relationships among island attributes, community structure and network architecture 29

Table S5. Summary of direct, indirect, and total effects for each response variable 30

Supplementary Methods 1:

Flower resource availability

We have no direct measure of pollen or nectar resources available per flower on different plant species, but we expect that greater numbers of flowers would equate to greater resources, and that larger flowers would have more resources than smaller flowers (all other things being equal). Therefore, we calculated species-specific estimates of the number and size (two-dimensional area) of flowers per individual plant per species, and summed these across the transect to obtain an estimate of total flower resource availability [1-4]. Although this measure will not be accurate in an absolute sense, we expect that it will scale proportionate to increasing floral resources across sites.

Estimating flower abundance and size (estimation methods differ for different plant species): (i) For herbs with dense flowering (e.g., *Erigeron annuus*, *Cnidium monnieri*, *Sinosenecio oldhamianus*), the ground cover area of the whole plant was estimated as the flower area by observation; (ii) For plants with sparse flowers (e.g. *Potentilla kleiniana*, *Lysimachia congestiflora*, *Hypericum sampsonii*), we projected the two-dimensional area of the petals onto a circle and approximated the area of the circle as the flower area (i.e., floral display size). This method also applies to plants with large flowers (such as *Rosa laevigata* and *Rosa bracteata*), that were straight-forward to count and measure flower area. This method is reasonable, since flower display size is related to pollinator attraction. (iii) For plants with long inflorescences (e.g., *Buddleja lindleyana*, *Leonurus artemisia*) or vines (e.g., *Millettia dielsiana*, *Wisteria sinensis*), the total inflorescence size was estimated as the surface area of a cylinder based on the radius and height of the inflorescence. (iv) For densely-flowering shrubs with small flowers (e.g., *Vitex*

negundo, *Symplocos paniculata*, *Photinia serrulata*), the crown surface area was estimated as the flower area, considering the crown of the shrub as a convex hull such as a sphere, cylinder or cone, as appropriate.

In a single survey, the observed floral display area of plants was estimated per species and then accumulated to obtain floral resource availability per transect, and then finally summed across multiple surveys. Therefore, the summed floral resource across multiple surveys represents the relative size of floral resource on the mainland and each island.

Supplementary Methods 2:

Sample completeness

We used sample coverage estimators to control for variation in the level of sampling completeness among sites. Sample coverage [5] uses species accumulation curves to estimate the probability that the next individual encountered belongs to a species already present in the community sample [6,7]. The principle is that based on the frequencies of species in the sample, the proportion of individuals belonging to undetected species can be estimated [8]. For plant-pollinator networks, sampling completeness typically focuses on both species richness [9] and interaction richness [10-12]. Here, we calculate sample coverage for plant species, pollinator species and plant-pollinator interactions using the *iNEXT* v.2.0.20 package [7] in R version 4.0.1 [13].

Supplementary Methods 3:

Species composition

Species composition on the mainland and 41 islands was examined by ordination analysis using non-metric multidimensional scaling (NMDS). NMDS identifies the dominant gradients of variation in site dissimilarity in ordination space (using Bray-Curtis distance in our case) and maps the rank ordering of sites onto a predefined small number of axes (k = 3) in an iterative search for an optimal solution [14, 15]. We used permutational multivariate analysis of variance (PERMANOVA) to test differences in species composition between edge and interior on the mainland and 41 islands. As a non-parametric multivariate test based on distances (again, Bray-Curtis distance in our case), PERMANOVA uses permutation to test the null hypothesis that the centroids and dispersion are equivalent for all groups [16]. NMDS and PERMANOVA were conducted using the *metaMDS* and *adonis2* functions in R package *vegan*, version 2.5-7 [17].

Furthermore, a basic checkerboard diagram is used to visually show how the relative specialisation vs. generalisation of plants and pollinators varies between edges vs. interiors. In the checkerboard diagram, rows and columns denote plant species and pollinator species, respectively. The red boxes represent the interactions occurring only at the island edge, dark blue boxes represent interactions occurring only in the island interior, and light blue boxes represent interactions occurring at both. Species in the checkerboard diagram are arranged from low specialization to high specialization (i.e., generalization decreases). We show the complete system-level meta-network by combining edge networks and interior networks of all 41 islands as well as the individual island-level meta-networks by combining the edge network and the interior network of each island separately. We draw the checkerboard diagram using R package *bipartite*, version 2.16 [18].

Supplementary Methods 4:

Causal hypotheses for the structural equation model

Habitat fragmentation can have both direct and indirect effects on community structure and network architecture, and there can be complex causal relationships among network attributes (Fig. 2). We based our hypothesized causal relationships on available theoretical and empirical findings from recent research, as detailed below.

Fragmentation has been shown to have strong direct effects on community structure. There is strong evidence that declining habitat area and increasing isolation have negative effects on plant abundance and richness on islands [19, 20]. Consequently, for island interior communities, we hypothesized that floral resources and plant richness might decrease with decreasing forest area and increasing isolation [21-24]. However, we may expect that habitat edges provide some degree of buffering of the negative effects of fragmentation on richness (an antagonistic interaction effect with area), because edges usually present a complex vertical vegetation structure, invasion of weedy second-growth plants is common [25-28], and factors such as high-intensity light conditions at edges are known to increase floral resources [29, 30]. Pollinators depend directly on the availability of floral resources [31-33]. Moreover, in previous studies, pollinator abundance and richness have been shown to decrease with increasing fragmentation [12, 34], while open habitats, such as forest edges, roads and powerline corridors tend to support diverse and abundant communities of pollinators [35-37]. Thus we hypothesized that pollinator abundance and richness would be high at island edges, particularly in large habitat areas, where floral resources are high.

Among the community-level variables measured, we hypothesized that floral resource availability and plant species richness would be positively correlated [38], but not necessarily causally related, per se. By contrast, high floral resource availability will tend to increase insect

pollinator abundance and richness [33], so we hypothesized that they are causally related. Meanwhile, for pollinator insects themselves, it is well recognised that high sample abundance of pollinators typically leads to higher pollinator richness [38], thus we hypothesized that they would be causally related.

Variation in plant-pollinator community structure can have cascading influences on network architecture [39-41]. Increasing plant and pollinator richness has generally been shown to decrease connectance and nestedness, but increase modularity [42-44]. By comparison, the opposite relationship might be expected for the effect of floral resource availability and pollinator abundance on network architecture, because with increasing abundance more potential interactions will be realized, which will tend to increase connectance and nestedness, but decrease modularity [34]. In keeping with these generally observed relationships, we hypothesized that an increase in plant and pollinator richness would potentially decrease connectance and nestedness, but increase modularity, while the opposite relationships would hold for floral resources and pollinator abundance. We note that these hypotheses would not be supported in cases where a highly compartmentalised network has strong boundaries between modules, since the addition of a new species would then not necessarily alter nestedness [45].

Among the measured network architecture variables, connectance is known to be positively associated with nestedness but negatively associated with modularity [34, 46, 47], so we hypothesized that increasing connectance would cause an increase in nestedness but a decrease in modularity. Nestedness and modularity are found to covary with one another in ecological networks [43, 48]. In mutualistic networks, nestedness and modularity have the opposite relationships, with nestedness promoting network stability whereas modularity reduces network stability, for instance. If one

increases the other is bound to decrease [40, 41, 49], so we hypothesized that they are negatively correlated in the SEM.

Different network attributes make different architectural contributions to network robustness to extinctions [41, 50]. As connectance increases, there are more possible interactions realized, which gives more chance for species to switch partners [51, 52], thus promoting network stability. The same relationship holds for nestedness, with more nested plant-pollinator networks allowing more species to coexist and providing more possibilities for interactions to happen, thus resulting in a more structurally stable network [40, 53, 54]. Conversely, high modularity tends to decrease the network robustness [41, 49]. Thus, we hypothesized that higher connectance and nestedness would lead to greater network robustness, while the reverse would hold true for modularity.

Lastly, some types of relationship have received little attention from researchers, particularly the potential for residual direct effects of fragmentation on network architecture that occur via mechanisms other than changing abundance or richness of plants and pollinators. For instance, temperature change could have direct effects on network connectance [55], there could be potential extinction of interactions due to phenological changes [56], or interactions may change due to temporal or spatial mismatches [57, 58]. Therefore, microclimate changes and complex spatial pattern caused by fragmentation may directly lead to changes in network architecture. We cannot predict the causal mechanisms, magnitude or direction of these effects in advance, so here we simply hypothesize that there are potential residual direct effects of forest area, proximity to edge, and isolation on network architecture.

Supplementary Methods 5:

Null model approach

A null model in ecology is an algorithm used to generate patterns that one might expect to occur at random in the absence of the hypothesized ecological mechanisms [59, 60]. In many cases null models are generated by simply re-sampling from the pool of observed data, with simulations constrained by underlying characteristics of individual samples [61, 62]. This approach has been used widely in testing non-random patterning in species interaction networks [63-67]. It is rarer for null model simulations to be used to generate expected patterns from external reference datasets, despite these being particularly useful in testing the effects of global environmental change [68]. In our research, the islands became isolated from continuous mainland habitats by creation of the TIL hydroelectric dam 60 years ago, so by using the adjacent mainland habitats to represent an unfragmented 'reference state' we are able to test re-assembly trajectories of plant-pollinator communities on islands over the course of habitat reduction and isolation. Intrinsically, sampling effort varied with forest area in the sampling design, potentially leading to confounding effects of sampling completeness on variation in species abundance and richness. Consequently, we constructed null model algorithms that draw random pollinator networks from the pooled 'reference' network at mainland sites (16 transects in the mainland edge: 52 plant species, 266 pollinator species, and 1098 individual interactions; and 16 transects in the mainland interior: 19 plant species, 92 pollinator species, and 152 individual interactions), constraining random draws by (I) the same number of transects used to acquire the observed floral resources, plant richness, pollinator abundance and pollinator richness on each island, and (II) the same number of pairwise interactions as well as the same numbers of plant and pollinator species observed on

each island (to account for the potential dependence of network architecture metrics on network size).

Null model I: Constraining to the same number of transects

For each of the 41 islands, we conducted a random draw of the same number of transects as sampled on the island (across the 20 sampling intervals), which is similar to the method of bootstrapping (i.e., in the case where a set of observations are assumed to be from an independent and identically distributed population), and then calculated cumulative floral resources, plant richness, pollinator abundance, and pollinator richness. This process was repeated 1000 times, generating the null expected mean value (± 95% confidence limits) for each variable from 1,000 null draws.

Null model II: Constraining both network abundance and network size

With respect to null model II, we refer to the originally proposed principle of network null models by Vázquez *et al*. [69], but with the difference that we considered forbidden and non-forbidden interactions. When we conducted null random draws of pairwise interactions, we only drew the non-forbidden links from the 'reference' pool. At the same time, interaction probabilities were determined by the relative abundances of plants and pollinators [69]. The re-sampling process was repeated 1000 times, generating 1000 simulated null networks for each island. We repeated this process separately for the edge and interior networks of each island. Note that interaction identities are not involved in the entire process of constructing the null model estimates of network structure, therefore it is not relevant whether particular interactions occur on islands which are not present in the mainland (or vice versa). The important issue is that sampling completeness is relatively high for the reference pool. The mainland reference pool has more

species and individuals than any individual island (Table S1), and sampling completeness is as high, or higher, than individual islands (Extended Data Fig.1), so in no case are we drawing a larger sample from a smaller pool.

In null model II we constrained null draws by both network abundance and network size. The simplest and most intuitive approach to null model II was a fully-random process in which potential plant and pollinator species were drawn at random until the number of species was equal to that of the observed network, after which potential interactions were drawn at random until equal to that of the observed network. Following the random draws, the resulting null network was evaluated to determine whether it was equal in size to the observed network (A1-A3 in Extended Data Fig.5). However, the probability of the null network meeting both network abundance and size criteria was low and the fully-random process was computationally slow, except for networks that contained only a small number of plant and pollinator species but had a high network abundance. Therefore, a more computationally efficient constrained 'stepwise-random' process was developed (B1-B4 in Extended Data Fig.5). First, following the random draw of potential plant species (network rows), the randomly-drawn pollinator species (network columns) were constrained to only those with known potential links in the reference pool, in order to avoid null network column totals that intrinsically sum to zero. Second, prior to the random draw of potential interactions, the null network was 'auto-populated' with the minimum number of randomly-drawn interactions to avoid network row and column totals stochastically summing to zero. Finally, after null network size was constrained to equal the observed network size, additional potential interactions were drawn at random to equal observed network abundance. More detailed information is provided below, using the comparatively simple

network for island S20 (Extended Data Fig.6) to illustrate the process of generating one random network draw from the mainland reference pool.

(A) Fully random process:

A1. For each of the 41 islands, we constructed a null 'expected' network by randomly drawing from the mainland reference pool, first, the same number of candidate plant species (weighting draws by the relative frequency of floral resources), and second, the same number of candidate pollinators (weighting draws by the relative frequency of pollinator abundance), as observed on the island (i.e., constraining network size). Using these species, we constructed a blank null draw network.

A2. For the resulting network, we then randomly added pairwise plant-pollinator interactions drawn from the potential (i.e., non-forbidden) links (those plants and pollinators contained in the mainland reference pool) at the relative frequencies expected from the mainland reference pool, until we reached the same number of pairwise interactions as observed on the respective islands.

A3. We evaluated the resulting null network against the logical expectation that all row and column totals should be greater than zero;

A3.1. If the null draw network had any row or column total equal to zero, we repeated the null draw process (A1-A3) until the expected network size was equal to the observed network size (Extended Data Fig.7a);

A3.2. If the null draw network had no row or column totals equal to zero, we accepted the null draw outcome (observed and expected network sizes and abundances were equal) (Extended Data Fig.7b).

(B) Stepwise-random process:

Although the null draw procedure described in steps A1-A3 is feasible in theory, it is computationally intensive in practice, particularly in highly asymmetrical networks with few plants and many pollinators (repeating at length at step A3.1). To maintain the philosophy of the random draw, but greatly speed up the algorithm, we added intermediate steps to derive a more efficient null draw process.

B1. For each of the 41 islands, we constructed a null 'expected' network by randomly drawing species and interactions from the mainland reference pool: first, we conducted a random draw of the same number of candidate plant species (weighting draws by the relative frequency of floral resources) as observed on the island; second, we constrained the reference pollinator pool to only the potential (i.e. non-forbidden) links expected for that particular candidate set of plants in the mainland reference pool; and third, from the constrained pool, we conducted a random draw of the same number of pollinators (weighting draws by the relative frequency of pollinator abundance) as observed on the island. This avoided pollinator column totals that might intrinsically sum to zero on every iteration of the algorithm.

B2. For the resulting candidate network, we auto-populated the network with all potential (i.e., non-forbidden) plant-pollinator interaction links known to occur in the mainland reference pool (regardless of their relative frequencies), in order to check for plant row totals that might stochastically sum to zero for that particular candidate set of pollinators.

B2.1. If the candidate null draw network had any row or column total equal to zero, we repeated the null draw process (B1-B2) until the expected network size was equal to the observed network size (Extended Data Fig.8a);

B2.2. If the candidate null draw network had no row or column totals equal to zero, we accepted the null draw outcome (observed and expected network sizes were equal) (Extended Data Fig.8b).

B3. For the resulting 'potential' network structure (with expected network size theoretically equal to observed), we randomly fixed one interaction link (based on the relative frequency of interaction abundance) in each row and column, respectively, thus ensuring the fewest interaction links necessary to constrain the network size (i.e., no row or column totals equal to zero). Three steps were used to achieve this goal (Extended Data Fig.8b):

B3.1 First, we randomly selected one interaction link to 'fix' in each row of the 'potential' null network, based on the link probabilities in the mainland reference pool. This produced a 'candidate' network in which each row total was equal to one.

B3.2 Second, we selected the network columns that still had column totals equal to zero after step B3.1, and randomly selected one interaction link to fix in these columns, based on the expected link probabilities in the mainland reference pool. This produced a 'candidate' network in which each row and column total was greater than or equal to one.

B3.3 Third, step B3.2 potentially created multiple links per network row. We checked and removed superfluous fixed links from rows, to acquire the best 'acceptable' network structure with all row and column totals greater than zero, and a minimum number of randomly-drawn fixed links.

B4. For the best 'acceptable' network, we then randomly added additional pairwise plant-pollinator interactions (up to the number observed on the island minus the number of fixed links

in B3). Interactions were drawn only from the non-forbidden links based on the expected link probabilities in the mainland reference pool. From this process, we acquired a null draw network that had the same network size as observed on the respective islands, as well as the same number of pairwise interactions (Extended Data Fig.8b).

We then calculated the network attributes (relative connectance, nestedness, modularity, robustness) and compared observed network attributes for each island against the mean expected value (± 95% confidence limits) from 1,000 null draws.

Supplementary Results 1:

Species composition at edge and interior of islands

Using a basic checkerboard diagram with species ranked based on network nestedness, we show visually how the relative specialisation vs. generalisation of plants and pollinators varies between edges vs. interiors (Extended Data Fig.4). We show the system-level meta-network by combining all edge and interior networks of all 41 islands (Extended Data Fig.4a) as well as the island-level meta-networks by combining the edge network and the interior network of each island separately (Extended Data Fig.4b). From both a system-level and an island-level meta-network perspective, the results in Extended Data Fig.4 show that most species in interior networks are not more specialized than species at edges, while the edge network simultaneously contains many specialists as well as generalists (not just one or the other). That is, island edges, in addition to having higher species richness, also contain highly specialized species.

Supplementary Results 2:

Sampling results at edge and interior of islands

In dense forested systems, such as this, the abundance of flowering plants in the interior is much lower than at the edge, so for the same level of survey effort the likelihood of detecting all the flowers along the transect is higher, and the available observation time per flower greater, in the interior than at the edge. Supplementary Table S1 shows that floral resources were approximately 18.52 times higher at the edge than in the interior, and pollinator abundance approximately 18.43 times higher at the edge. These data are from 116 pairs of edge vs. interior transects surveyed 20 times each over 3 years, using the 15-minute survey interval for edge transects and 10-minute survey interval for interior transects. The 1.5 times difference in survey

interval cannot account for the very large difference in observed detections. We can show this quantitatively using sample accumulation curves (Extended Data Fig.2), scaled by number of survey hours on the x-axis, emphasizing the dramatically lower encounter rate of flower-pollinator interactions per hour of cumulative survey time in the island interior.

Reference

1. Vazquez, Diego, P., Dorado & Jimena The diversity-stability relationship in floral production. *Oikos* **123**, 1137-1143 (2014).
2. Weiner, C.N., Werner, M., Linsenmair, K.E. & Blüthgen, N. Land-use impacts on plant-pollinator networks: interaction strength and specialization predict pollinator declines. *Ecology* **95**, 466-474 (2016).
3. Sutter, L. *et al.* Enhancing plant diversity in agricultural landscapes promotes both rare bees and dominant crop-pollinating bees through complementary increase in key floral resources. *J Appl Ecol* **54**, 1856-1864 (2017).
4. Adedoja, O., Dormann, C.F., Kehinde, T. & Samways, M.J. Refuges from fire maintain pollinator-plant interaction networks. *Ecol. Evol.* **9**, 5777-5786 (2019).
5. Chao, A. *et al.* Rarefaction and extrapolation with Hill numbers: a framework for sampling and estimation in species diversity studies. *Ecol Monogr* **84**, 45-67 (2014).
6. Roswell, M., Dushoff, J. & Winfree, R. A conceptual guide to measuring species diversity. *Oikos* **130**, 321-338 (2021).
7. Hsieh, T.C., Ma, K.H. & Chao, A. iNEXT: an R package for rarefaction and extrapolation of species diversity (Hill numbers). *Methods Ecol. Evol.* **7**, 1451-1456 (2016).
8. Chao, A. & Jost, L. Coverage-based rarefaction and extrapolation: standardizing samples by completeness rather than size. *Ecology* **93**, 2533-2547 (2012).
9. Goldwasser, L. & Roughgarden, J. Sampling effects and the estimation of food-web properties. *Ecology* **78**, 41-54 (1997).
10. Chacoff, N.P. *et al.* Evaluating sampling completeness in a desert plant-pollinator network. *J Anim Ecol* **81**, 190-200 (2012).
11. Devoto, M., Bailey, S., Craze, P. & Memmott, J. Understanding and planning ecological restoration of plant-pollinator networks. *Ecol. Lett.* **15**, 319-328 (2012).
12. Grass, I., Jauker, B., Steffan-Dewenter, I., Tscharntke, T. & Jauker, F. Past and potential future effects of habitat fragmentation on structure and stability of plant-pollinator and host-parasitoid networks. *Nat. Ecol. Evol.* **2**, 1408-1417 (2018).
13. R Core Team. R: A Language and Environment for Statistical Computing (R Foundation for Statistical Computing, 2020).
14. Shepard, R. The analysis of proximities: Multidimensional scaling with an unknown distance function. I. *Psychometrika* **27**, 125-140 (1962).
15. Kruskal, J. Nonmetric multidimensional scaling: A numerical method. *Psychometrika* **29**, 115-129 (1964).
16. Anderson, M.J. A new method for non-parametric multivariate analysis of variance. *Austral Ecol* **26**, 32-46 (2001).
17. Oksanen, J. *et al.* vegan: Community ecology package. R package version 2.5-7 https://CRAN.R-project.org/package=vegan (2020).
18. Dormann, C.F., Fruend, J., Gruber, B., Dormann, M.C.F., LazyData, T. & ByteCompile, T. bipartite: Visualising bipartite networks and calculating some (ecological) indices. R package version 2.16 https://CRAN.R-project.org/package=bipartite (2021).
19. Gardner, R.H. & Engelhardt, K.A.M. Spatial processes that maintain biodiversity in plant communities. *Perspect. Plant Ecol. Evol. Syst.* **9**, 211-228 (2008).
20. Keppel, G., Buckley, Y.M. & Possingham, H.P. Drivers of lowland rain forest community assembly, species diversity and forest structure on islands in the tropical South Pacific. *J. Ecol.* **98**, 87-95 (2010).
21. Smith, M.D. & Knapp, A.K. Dominant species maintain ecosystem function with non-random species loss. *Ecol. Lett.* **6**, 509-517 (2003).

22. Rezende, E.L., Lavabre, J.E., Guimaraes, P.R., Jordano, P. & Bascompte, J. Non-random coextinctions in phylogenetically structured mutualistic networks. *Nature* **448**, 925-928 (2007).
23. Wardle, D.A., Bardgett, R.D., Callaway, R.M. & Van der Putten, W.H. Terrestrial ecosystem responses to species gains and losses. *Science* **332**, 1273-1277 (2011).
24. May, F., Rosenbaum, B., Schurr, F.M. & Chase, J.M. The geometry of habitat fragmentation: Effects of species distribution patterns on extinction risk due to habitat conversion. *Ecol. Evol.* **9**, 2775-2790 (2019).
25. Didham, R.K. & Lawton, J.H. Edge structure determines the magnitude of changes in microclimate and vegetation structure in tropical forest fragments. *Biotropica* **31**, 17-30 (1999).
26. Honnay, O., Verheyen, K. & Hermy, M. Permeability of ancient forest edges for weedy plant species invasion. *For. Ecol. Manag.* **161**, 109-122 (2002).
27. Ries, L., Fletcher, R.J., Battin, J. & Sisk, T.D. Ecological responses to habitat edges: Mechanisms, models, and variability explained. *Annu Rev Ecol Evol Syst* **35**, 491-522 (2004).
28. Erdos, L. *et al.* Plant composition and diversity at edges in a semi-natural forest-grassland mosaic. *Plant Ecol* **220**, 279-292 (2019).
29. Ries, L. & Sisk, T.D. A predictive model of edge effects. *Ecology* **85**, 2917-2926 (2004).
30. Harper, K.A. *et al.* Edge influence on forest structure and composition in fragmented landscapes. *Conserv. Biol.* **19**, 768-782 (2005).
31. Sih, A. & Baltus, M.S. Patch size, pollinator behavior, and pollinator limitation in Catnip. *Ecology* **68**, 1679-1690 (1987).
32. Fründ, J., Linsenmair, K.E. & Bluthgen, N. Pollinator diversity and specialization in relation to flower diversity. *Oikos* **119**, 1581-1590 (2010).
33. Hadley, A.S. & Betts, M.G. The effects of landscape fragmentation on pollination dynamics: absence of evidence not evidence of absence. *Biol. Rev.* **87**, 526-544 (2012).
34. Spiesman, B.J. & Inouye, B.D. Habitat loss alters the architecture of plant-pollinator interaction networks. *Ecology* **94**, 2688-2696 (2013).
35. Hopwood, J.L. The contribution of roadside grassland restorations to native bee conservation. *Biol. Conserv.* **141**, 2632-2640 (2008).
36. Wojcik, V.A. & Buchmann, S. Pollinator conservation and management on electrical transmission and roadside rights-of-way: a review. *J. Pollinat. Ecol.* **7**, 16-26 (2012).
37. Bailey, S. *et al.* Distance from forest edge affects bee pollinators in oilseed rape fields. *Ecol. Evol.* **4**, 370-380 (2014).
38. Woodcock, B.A. *et al.* Meta-analysis reveals that pollinator functional diversity and abundance enhance crop pollination and yield. *Nat. Commun.* **10**, 1-10 (2019).
39. Fortuna, M.A. & Bascompte, J. Habitat loss and the structure of plant-animal mutualistic networks. *Ecol. Lett.* **9**, 278-283 (2006).
40. Okuyama, T. & Holland, J.N. Network structural properties mediate the stability of mutualistic communities. *Ecol. Lett.* **11**, 208-216 (2008).
41. Thebault, E. & Fontaine, C. Stability of ecological communities and the architecture of mutualistic and trophic networks. *Science* **329**, 853-856 (2010).
42. Dunne, J.A., Williams, R.J. & Martinez, N.D. Network structure and biodiversity loss in food webs: robustness increases with connectance. *Ecol. Lett.* **5**, 558-567 (2002).
43. Olesen, J.M., Bascompte, J., Dupont, Y.L. & Jordano, P. The modularity of pollination networks. *Proc. Natl. Acad. Sci. U.S.A.* **104**, 19891-19896 (2007).
44. Campbell, C., Yang, S.A., Albert, R. & Shea, K. Plant-pollinator community network response to species invasion depends on both invader and community characteristics. *Oikos* **124**, 406-413 (2015).
45. Cai, W., Snyder, J., Hastings, A. & D'Souza, R.M. Mutualistic networks emerging from adaptive niche-based interactions. *Nat. Commun.* **11**, 1-10 (2020).
46. Vieira, M.C. & Almeida-Neto, M. A simple stochastic model for complex coextinctions in mutualistic networks: robustness decreases with connectance. *Ecol. Lett.* **18**, 144-152 (2015).

47. Valdovinos, F.S. *et al.* Niche partitioning due to adaptive foraging reverses effects of nestedness and connectance on pollination network stability. *Ecol. Lett.* **19**, 1277-1286 (2016).
48. Fortuna, M.A. *et al.* Nestedness versus modularity in ecological networks: two sides of the same coin? *J Anim Ecol* **79**, 811-817 (2010).
49. Morrison, B.M. & Dirzo, R. Distinct responses of antagonistic and mutualistic networks to agricultural intensification. *Ecology* **101**, e03116 (2020).
50. Ramos-Jiliberto, R., Valdovinos, F.S., Moisset de Espanés, P. & Flores, J.D. Topological plasticity increases robustness of mutualistic networks. *J Anim Ecol* **81**, 896-904 (2012).
51. Evans, D.M., Pocock, M.J.O. & Memmott, J. The robustness of a network of ecological networks to habitat loss. *Ecol. Lett.* **16**, 844-852 (2013).
52. Ponisio, L.C., Gaiarsa, M.P. & Kremen, C. Opportunistic attachment assembles plant-pollinator networks. *Ecol. Lett.* **20**, 1261-1272 (2017).
53. Pawar, S. Why are plant-pollinator networks nested? *Science* **345**, 383-383 (2014).
54. Rohr, R.P., Saavedra, S. & Bascompte, J. On the structural stability of mutualistic systems. *Science* **345**, 1253497 (2014).
55. Petchey, O.L., Brose, U. & Rall, B.C. Predicting the effects of temperature on food web connectance. *Philos. Trans. R. Soc. Lond., B, Biol. Sci.* **365**, 2081-2091 (2010).
56. Memmott, J., Craze, P.G., Waser, N.M. & Price, M.V. Global warming and the disruption of plant-pollinator interactions. *Ecol. Lett.* **10**, 710-717 (2007).
57. Burkle, L.A., Marlin, J.C. & Knight, T.M. Plant-pollinator interactions over 120 years: loss of species, co-occurrence, and function. *Science* **339**, 1611-1615 (2013).
58. Hegland, S.J., Nielsen, A., Lázaro, A., Bjerknes, A.L. & Totland, Ø. How does climate warming affect plant-pollinator interactions? *Ecol. Lett.* **12**, 184-195 (2009).
59. Gotelli, N.J. & Graves, G.R. *Null models in ecology*. (Smithsonian Inst. Press, 1996).
60. Farine, D.R. & Whitehead, H. Constructing, conducting and interpreting animal social network analysis. *J Anim Ecol* **84**, 1144-1163 (2015).
61. Gotelli, N.J. & Ulrich, W. Statistical challenges in null model analysis. *Oikos* **121**, 171-180 (2012).
62. Farine, D.R. A guide to null models for animal social network analysis. *Methods Ecol. Evol.* **8**, 1309-1320 (2017).
63. Ulrich, W. & Gotelli, N.J. Null model analysis of species nestedness patterns. *Ecology* **88**, 1824-1831 (2007).
64. Blüthgen, N., Fründ, J., Vázquez, D.P. & Menzel, F. What do interaction network metrics tell us about specialization and biological traits. *Ecology* **89**, 3387-3399 (2008).
65. Dormann, C.F. & Strauss, R. A method for detecting modules in quantitative bipartite networks. *Methods Ecol. Evol.* **5**, 90-98 (2014).
66. Strona, G., Nappo, D., Boccacci, F., Fattorini, S. & San-Miguel-Ayanz, J. A fast and unbiased procedure to randomize ecological binary matrices with fixed row and column totals. *Nat. Commun.* **5**, 1-9 (2014).
67. Vaughan, I.P. *et al.* econullnetr: an R package using null models to analyse the structure of ecological networks and identify resource selection. *Methods Ecol. Evol.* **9**, 728-733 (2018).
68. Schafer, R.B. & Piggott, J.J. Advancing understanding and prediction in multiple stressor research through a mechanistic basis for null models. *Glob Chang Biol* **24**, 1817-1826 (2018).
69. Vázquez, D.P. *et al.* Species abundance and asymmetric interaction strength in ecological networks. *Oikos* **116**, 1120-1127 (2007).

Table S1. Site attributes for the mainland and 41 island sites, with mean plant and pollinator community attributes for transects at edge and interior locations over 3 years sampling combined. Floral resources represent cumulative cover of flowers (in m^2) across all 20 survey intervals combined, as a measure of floral resource availability at each site.

**Site Code**	**Island area (ha)**	**Distance to mainland (m)**	**Number of paired transect**	**Average edge to interior difference in elevation (m)**	**Edge transects**				**Interior transects**			
					Floral resources	Plant richness	Pollinator richness	Pollinator abundance	Floral resources	Plant richness	Pollinator richness	Pollinator abundance
Mainland	-	-	16	43.62	1611.20	52	266	3692	70.80	19	91	235
B01	1158.09	884.48	16	41.05	1471.80	42	260	3177	87.80	18	87	177
B02	128.04	1452.28	9	43.50	703.54	44	208	1475	32.46	14	48	72
B03	31.00	2134.78	4	48.60	119.62	23	127	561	10.18	8	33	42
B04	36.56	197.01	4	37.75	189.10	26	146	830	10.30	9	32	48
B05	101.02	964.61	9	45.00	423.94	30	157	902	9.86	9	33	56
B06	51.89	950.35	4	38.85	188.16	29	158	740	7.24	8	29	52
B07	29.05	1938.73	4	39.05	169.72	28	146	717	9.48	8	23	35
S01	0.43	3725.02	1	13.00	40.54	21	67	220	2.86	5	12	14
S02	0.62	3204.96	1	13.10	42.40	19	86	318	3.20	5	12	12
S03	1.42	3359.06	2	14.80	73.34	17	97	379	4.66	5	12	13
S04	1.16	3547.04	2	17.70	30.08	13	66	351	0.92	5	9	11
S05	0.59	2320.77	1	19.50	8.58	11	25	66	1.02	5	5	8
S06	0.63	2127.51	1	16.90	13.64	10	59	120	1.16	4	7	9
S07	0.49	2105.77	1	17.80	21.48	12	68	144	1.32	5	7	9
S09	1.82	1046.34	2	19.80	54.40	15	52	112	9.20	3	5	6
S10	1.38	851.24	2	14.10	32.80	9	38	99	7.20	6	9	10
S11	1.56	1098.86	2	23.40	27.24	10	42	85	1.96	3	5	6
S14	0.42	665.66	1	12.30	23.00	16	63	176	3.10	5	6	6
S16	0.26	724.95	1	9.00	16.10	7	39	128	0.30	1	2	2
S17	0.71	892.70	1	21.00	21.60	15	75	267	2.40	5	6	6
S18	0.41	1184.77	1	9.80	7.50	6	26	51	0.50	2	3	4
S20	1.28	928.40	2	19.40	44.28	16	75	258	3.52	5	7	11
S21	0.57	1129.17	1	20.70	10.20	7	42	93	2.20	3	3	4
S22	0.65	1370.34	1	22.20	11.04	9	29	109	0.36	2	3	3
S23	1.51	787.69	2	28.15	29.00	9	55	214	2.80	3	4	4
S24	0.17	1339.86	1	12.20	9.00	8	34	90	1.00	3	3	3
S25	0.73	1776.70	1	15.90	49.60	19	126	453	2.60	8	16	19
S26	9.73	2163.77	3	35.10	237.30	19	123	451	6.50	6	18	22
S27	0.53	2362.96	1	6.80	18.76	9	72	176	3.04	4	7	8
S35	0.34	3044.01	1	14.60	13.14	12	47	109	1.66	4	6	8
S37	3.03	2576.17	2	17.75	44.48	11	75	266	1.12	4	5	5
S38	0.92	2470.85	1	18.00	19.56	8	88	269	1.04	3	6	7
S39	0.98	1426.71	1	16.60	9.78	13	40	101	1.52	4	8	9
S40	1.43	1226.33	2	23.75	22.86	11	51	132	3.14	3	10	11
S41	1.19	1076.74	2	15.00	27.84	15	71	190	2.96	4	11	11
S42	3.03	1048.62	2	20.55	48.02	20	75	217	4.44	5	12	12
S43	2.23	1187.39	2	24.45	22.32	11	75	219	3.68	6	8	8
S44	1.35	984.41	2	24.55	33.94	12	67	218	1.10	2	6	8
S46	1.03	727.55	2	18.00	29.84	12	76	188	2.16	4	8	8
S47	0.29	1001.64	1	8.70	11.72	11	32	81	0.48	3	3	3
S48	0.89	356.09	1	25.10	9.24	15	25	39	0.36	3	5	6
Total	-	-	116	-	5991.70	68	313	18,483	323.60	31	175	1,003

Table S2. List of plant species observed and their cumulative floral resource availability (m^2) after 20 surveys in 3 years. Floral resource values are presented as combined totals across all 41 islands and mainland sites over 3 years. The 68 plant species belonged to 32 families and 58 genera, and the identification rate to species was 95.59%.

Family	Species	Code	Cumulative area of floral resources (m^2)	
			Edge	Interior
Apiaceae	*Cnidium monnieri* (L.) Cusson	PL1	426.48	26.32
Asteraceae	*Crepidiastrum sonchifolium* (Maxim.) Pak	PL2	24.40	0.00
Asteraceae	*Erigeron annuus* (L.) Pers.	PL3	310.50	25.50
Asteraceae	*Hemistepta lyrata* (Bunge) Bunge	PL4	70.90	0.20
Asteraceae	*Pseudognaphalium affine* (D.Don) Anderb.	PL5	72.20	0.00
Asteraceae	*Sinosenecio oldhamianus* (Maxim.)	PL6	309.56	16.44
Boraginaceae	*Trigonotis peduncularis* (Trevir.) Steven ex	PL7	5.80	0.00
Brassicaceae	*Brassica napus* L.	PL8	12.80	0.00
Brassicaceae	*Rorippa indica (*L.) Hiern	PL9	101.80	0.60
Caprifoliaceae	*Abelia × grandiflora* (Andre) Rehd	PL10	7.00	0.00
Caprifoliaceae	*Lonicera japonica* Thunb.	PL11	59.20	0.00
Clusiaceae	*Hypericum sampsonii* Hance	PL12	61.00	0.00
Convolvulaceae	*Ipomoea nil* (L.) Roth	PL13	28.20	0.00
Crassulaceae	*Sedum bulbiferum* Makino	PL14	7.00	0.00
Ericaceae	*Lyonia ovalifolia* (Wall.) Drude	PL15	3.40	0.80
Ericaceae	*Rhododendron ovatum* (Lindl.) Planch. ex	PL16	91.68	9.72
Ericaceae	*Rhododendron simsii* Planch.	PL17	20.24	18.36
Ericaceae	*Vaccinium bracteatum* Thunb.	PL18	12.78	10.42
Ericaceae	*Vaccinium* sp.	PL19	14.10	23.90
Euphorbiaceae	*Glochidion puberum* (L.) Hutch.	PL20	16.30	11.10
Euphorbiaceae	*Triadica sebifera* (L.) Small	PL21	279.60	0.00
Fabaceae	*Dalbergia hupeana* Hance	PL22	82.08	6.72
Fabaceae	*Indigofera tinctoria* L.	PL23	66.00	5.00
Fabaceae	*Lespedeza* sp.	PL24	1.00	14.40
Fabaceae	*Millettia dielsiana* Harms	PL25	130.20	0.00
Fabaceae	*Wisteria sinensis* (Sims) Sweet	PL26	37.60	0.00
Hamamelidaceae	*Loropetalum chinense* (R. Br.) Oliv.	PL27	0.60	7.60
Labiatae	*Leonurus artemisia* (Lour.) S.Y.Hu	PL28	566.80	2.80
Labiatae	*Salvia miltiorrhiza* Bunge	PL29	15.40	1.00
Labiatae	*Salvia plebeia* R.Br.	PL30	86.80	0.00
Labiatae	*Scutellaria barbata* D.Don	PL31	0.60	0.00
Labiatae	*Stachys japonica* Miq.	PL32	43.60	0.00
Liliaceae	*Liriope spicata* Lour.	PL33	6.00	1.20
Loganiaceae	*Buddleja lindleyana* Fortune	PL34	98.80	0.00
Oleaceae	*Ligustrum quihoui* Carrière	PL35	54.26	1.14
Oxalidaceae	*Oxalis corniculata* L.	PL36	8.40	0.00
Papaveraceae	*Corydalis pallida* (Thunb.) Pers.	PL37	3.80	8.40
Papaveraceae	*Macleaya cordata* (Willd.) R.Br.	PL38	132.20	0.00
Phytolaccaceae	*Phytolacca americana* L.	PL39	61.60	0.00
Polygalaceae	*Polygala tenuifolia* Willd.	PL40	15.60	0.00
Primulaceae	*Lysimachia congestiflora* Hemsl.	PL41	39.80	15.40
Primulaceae	*Lysimachia fortunei* Maxim.	PL42	288.86	15.14
Ranunculaceae	*Clematis florida* Thunb.	PL43	6.40	0.00
Ranunculaceae	*Ranunculus* sp.	PL44	27.00	0.00
Rhamnaceae	*Rhamnus crenata* Siebold & Zucc.	PL45	49.88	3.12
Rosaceae	*Photinia serrulata* auct.	PL46	26.00	8.40

Rosaceae	*Potentilla kleiniana* Wight & Arn.	PL47	62.60	0.00
Rosaceae	*Rhaphiolepis indica* (L.) Lindl.	PL48	0.82	2.38
Rosaceae	*Rosa bracteata* J.C.Wendl.	PL49	364.60	0.00
Rosaceae	*Rosa laevigata* Michx.	PL50	256.00	0.00
Rosaceae	*Rosa multiflora* Thunb.	PL51	129.60	0.00
Rosaceae	*Rubus corchorifolius* L.f.	PL52	96.20	0.00
Rosaceae	*Rubus coreanus* Miq.	PL53	33.60	0.00
Rosaceae	*Rubus hirsutus* Thunb.	PL54	213.00	0.00
Rosaceae	*Rubus sumatranus* Miq.	PL55	7.20	0.00
Rosaceae	*Spiraea salicifolia* L.	PL56	11.60	0.00
Rubiaceae	*Gardenia jasminoides* J.Ellis	PL57	188.66	2.74
Rubiaceae	*Paederia foetida* L.	PL58	26.20	0.00
Rubiaceae	*Serissa serissoides* (DC.) Druce	PL59	2.30	11.20
Saururaceae	*Houttuynia cordata* Thunb.	PL60	9.40	0.00
Scrophulariaceae	*Mazus japonicus* (Thunb.) Kuntze	PL61	17.50	0.00
Scrophulariaceae	*Monochasma savatieri* Franch. ex Maxim.	PL62	0.80	0.00
Solanaceae	*Solanum nigrum* L.	PL63	28.40	0.00
Symplocaceae	*Symplocos paniculata* (Thunb.) Miq.	PL64	19.06	15.34
Symplocaceae	*Symplocos sumuntia* Buch.-Ham. ex D.	PL65	163.82	21.98
Theaceae	*Schima superba* Gardner & Champ.	PL66	11.20	0.40
Verbenaceae	*Vitex negundo* L.	PL67	459.32	35.88
Vitaceae	*Cayratia japonica* (Thunb.) Gagnep.	PL68	105.60	0.00

Table S3. List of pollinators observed and their cumulative abundance at all sites over 3 years. The pollinators belonged to 5 orders, 70 families, 185 genera, and the identification rate was 58.47% (183 species) to species, 30.03% (94) to genus, 11.50% (36) only to family.

Order	Family	Species	Code	Pollinator abundance	
				Edge	Interior
Coleoptera	Anthicidae	Anthicidae sp.	PO1	27	0
Coleoptera	Attelabidae	Attelabidae sp.	PO2	8	0
Coleoptera	Buprestidae	*Anthaxia chinensis* Kerremans, 1898	PO3	46	0
Coleoptera	Buprestidae	*Anthaxia* sp.	PO4	75	19
Coleoptera	Cantharidae	*Cantharis nigricans* (O. F. Müller, 1776)	PO5	13	1
Coleoptera	Cantharidae	*Cantharis* sp.1	PO6	41	0
Coleoptera	Cantharidae	*Cantharis* sp.2	PO7	16	0
Coleoptera	Cantharidae	*Rhagonycha fulva* (Scopoli, 1763)	PO8	1	0
Coleoptera	Cantharidae	*Themus* sp.	PO9	1	0
Coleoptera	Cerambycidae	*Chlorophorus kanekoi* Matsushita, 1941	PO10	2	0
Coleoptera	Cerambycidae	*Purpuricenus spectabilis* Motschulsky, 1857	PO11	45	6
Coleoptera	Cerambycidae	*Strangalia fortunei* Pascoe, 1858	PO12	21	0
Coleoptera	Chrysomelidae	*Altica birmanensis* (Jacoby, 1896)	PO13	11	0
Coleoptera	Chrysomelidae	*Aulacophora indica* (Gmelin, 1790)	PO14	8	1
Coleoptera	Chrysomelidae	*Aulacophora lewisii* Baly, 1886	PO15	22	0
Coleoptera	Chrysomelidae	Chrysomelidae sp.1	PO16	33	4
Coleoptera	Chrysomelidae	Chrysomelidae sp.2	PO17	41	4
Coleoptera	Chrysomelidae	Chrysomelidae sp.3	PO18	57	0
Coleoptera	Chrysomelidae	*Hemipyxis flaviabdominalis* Chujo, 1965	PO19	11	0
Coleoptera	Chrysomelidae	*Longitarsus* sp.	PO20	91	1
Coleoptera	Chrysomelidae	*Nonarthra variabilis* Baly, 1862	PO21	23	1
Coleoptera	Chrysomelidae	*Paleosepharia liquidambara* Gressitt & Kimoto, 1963	PO22	109	1
Coleoptera	Chrysomelidae	*Phola octodecimguttata* (Fabricius, 1775)	PO23	17	4
Coleoptera	Chrysomelidae	*Phyllotreta* sp.	PO24	13	0
Coleoptera	Chrysomelidae	*Sphaeroderma* sp.1	PO25	19	2
Coleoptera	Cleridae	*Trichodes sinae* Chevr., 1874	PO26	13	0
Coleoptera	Coccinellidae	*Calvia muiri* (Timberlake, 1943)	PO27	9	0
Coleoptera	Coccinellidae	*Coccinella septempunctata* Linnaeus, 1758	PO28	329	7
Coleoptera	Coccinellidae	*Epilachna* sp.	PO29	14	0
Coleoptera	Coccinellidae	*Harmonia axyridis* (Pallas, 1773)	PO30	13	2
Coleoptera	Coccinellidae	*Lemnia saucia* (Mulsant, 1850)	PO31	6	0
Coleoptera	Coccinellidae	*Propylea japonoca* (Thunberg, 1780)	PO32	7	0
Coleoptera	Curculionidae	Curculionidae sp.1	PO33	18	0
Coleoptera	Curculionidae	Curculionidae sp.2	PO34	53	4
Coleoptera	Curculionidae	Curculionidae sp.3	PO35	25	1
Coleoptera	Curculionidae	*Piezotrachelus japonicus* (Roelofs, 1874)	PO36	154	13
Coleoptera	Elateridae	Elateridae sp.	PO37	25	4
Coleoptera	Elateridae	*Procraerus ligatus* (Candèze, 1878)	PO38	184	11
Coleoptera	Eumolpidae	*Smaragdina nigrifrons* (Hope, 1842)	PO39	8	0
Coleoptera	Eumolpidae	*Smaragdina* sp.	PO40	5	0
Coleoptera	Lagriidae	Lagriidae sp.1	PO41	60	2
Coleoptera	Lagriidae	Lagriidae sp.2	PO42	50	1
Coleoptera	Melyridae	*Attalus* sp.	PO43	22	5
Coleoptera	Melyridae	*Idgia flavicollis* Redtenbacher, 1868	PO44	65	0
Coleoptera	Melyridae	*Intybia* sp.	PO45	20	0
Coleoptera	Mordellidae	Mordellidae sp.1	PO46	2	0
Coleoptera	Mordellidae	Mordellidae sp.2	PO47	19	2
Coleoptera	Mordellidae	Mordellidae sp.3	PO48	20	0

Coleoptera	Nitidulidae	*Epuraea aestiva* (Linnaeus, 1758)	PO49	3	0
Coleoptera	Nitidulidae	*Melighetes* sp.	PO50	24	1
Coleoptera	Oedemeridae	Oedemeridae sp.	PO51	6	1
Coleoptera	Phalacridae	Phalacridae sp.	PO52	169	19
Coleoptera	Rutelidae	*Anomala corpulenta* Motschulsky, 1854	PO53	3	0
Coleoptera	Rutelidae	*Anomala coxalis* Bates, 1891	PO54	50	1
Coleoptera	Scarabaeidae	*Glycyphana fulvistemma* Motschulsky, 1858	PO55	1	0
Coleoptera	Scarabaeidae	*Hybovalgus major* Sawada, 1939	PO56	6	0
Coleoptera	Scarabaeidae	*Lasiotrichius succinctus* (Pallas, 1781)	PO57	60	0
Coleoptera	Scarabaeidae	*Paratrichius* sp.	PO58	104	2
Coleoptera	Staphylinidae	*Paederus fuscipes* Curtis, 1826	PO59	336	17
Diptera	Agromyzidae	Agromyzidae sp.1	PO60	174	15
Diptera	Agromyzidae	Agromyzidae sp.2	PO61	40	10
Diptera	Anthomyiidae	Anthomyiidae sp.	PO62	20	1
Diptera	Bibionidae	*Bibio tenebrosus* Coquillett, 1898	PO63	27	2
Diptera	Bibionidae	*Penthetria* sp.	PO64	55	3
Diptera	Bombyliidae	*Systropus* sp.1	PO65	42	1
Diptera	Bombyliidae	*Systropus* sp.2	PO66	3	0
Diptera	Bombyliidae	*Systropus* sp.3	PO67	5	0
Diptera	Calliphoridae	*Calliphoridae* sp.	PO68	7	0
Diptera	Calliphoridae	*Chrysomya megacephala* (Fabricius, 1794)	PO69	51	3
Diptera	Calliphoridae	*Chrysomya* sp.	PO70	41	4
Diptera	Calliphoridae	*Lucilia illustris* (Meigen, 1826)	PO71	12	0
Diptera	Calliphoridae	*Lucilia sericata* (Meigen, 1826)	PO72	13	3
Diptera	Calliphoridae	*Lucilia sinensis* Aubertin, 1933	PO73	14	0
Diptera	Calliphoridae	*Lucilia* sp.1	PO74	297	15
Diptera	Calliphoridae	*Lucilia* sp.2	PO75	25	2
Diptera	Celyphidae	Celyphidae sp.	PO76	4	1
Diptera	Drosophilidae	*Drosophila* sp.1	PO77	138	7
Diptera	Drosophilidae	*Drosophila* sp.2	PO78	311	16
Diptera	Lauxaniidae	Lauxaniidae sp.1	PO79	286	32
Diptera	Lauxaniidae	Lauxaniidae sp.2	PO80	83	8
Diptera	Milichiidae	Milichiidae sp.	PO81	65	5
Diptera	Muscidae	*Helicophagella melanura* Meigen, 1826	PO82	7	1
Diptera	Muscidae	*Helicophagella misera* (Walker, 1849)	PO83	282	28
Diptera	Muscidae	*Helicophagella* sp.1	PO84	29	2
Diptera	Muscidae	*Helicophagella* sp.2	PO85	76	15
Diptera	Muscidae	*Helicophagella* sp.3	PO86	69	3
Diptera	Muscidae	*Musca domestica* Linnaeus, 1758	PO87	38	2
Diptera	Sciomyzidae	*Sepedon* sp.	PO88	6	0
Diptera	Sepsidae	Sepsidae sp.1	PO89	73	11
Diptera	Sepsidae	Sepsidae sp.2	PO90	96	3
Diptera	Stratiomyidae	*Hermetia illucens* (Linnaeus, 1758)	PO91	14	0
Diptera	Stratiomyidae	*Hybos* sp.1	PO92	12	1
Diptera	Stratiomyidae	*Hybos* sp.2	PO93	4	0
Diptera	Stratiomyidae	*Philopoda nigroaenea*	PO94	5	0
Diptera	Stratiomyidae	*Syneches* sp.	PO95	5	0
Diptera	Syrphidae	*Asarkina porcina* (Coquillett, 1898)	PO96	8	0
Diptera	Syrphidae	*Episyrphus balteatus* (De Geer, 1776)	PO97	8	0
Diptera	Syrphidae	*Eristalinus arvorum* (Fabricius, 1787)	PO98	2	0
Diptera	Syrphidae	*Eristalinus* sp.	PO99	53	9
Diptera	Syrphidae	*Eristalis* sp.1	PO100	32	4
Diptera	Syrphidae	*Eristalis* sp.2	PO101	29	3
Diptera	Syrphidae	*Eristalis tenax* (Linnaeus, 1758)	PO102	10	2
Diptera	Syrphidae	*Helophilus virgatus* (Coquilletti, 1898)	PO103	38	1
Diptera	Syrphidae	*Melanostoma scalare* (Fabricius, 1794)	PO104	46	0
Diptera	Syrphidae	*Monoceromyia* sp.	PO105	3	0
Diptera	Syrphidae	*Paragus haemorrhous* Meigen, 1822	PO106	16	0
Diptera	Syrphidae	*Phytomia errans* (Fabricius, 1787)	PO107	3	0
Diptera	Syrphidae	*Phytomia zonata* (Fabricius, 1787)	PO108	17	0

Diptera	Syrphidae	*Pipiza inornata* Matsumura, 1916	PO109	67	3
Diptera	Syrphidae	*Platycheirus* sp.	PO110	36	2
Diptera	Syrphidae	*Sphaerophoria indiana* Bigot, 1884	PO111	28	1
Diptera	Syrphidae	*Sphaerophoria menthastri* (Linnaeus, 1758)	PO112	57	5
Diptera	Syrphidae	*Sphaerophoria rueppelli* (Wiedemann, 1830)	PO113	14	0
Diptera	Syrphidae	*Sphaerophoria scripta* (Linnaeus, 1758)	PO114	16	0
Diptera	Syrphidae	*Sphaerophoria* sp.	PO115	5	1
Diptera	Syrphidae	*Sphaerophoria taeniata* (Meigen, 1822)	PO116	11	0
Diptera	Syrphidae	*Sphiximorpha* sp.	PO117	37	1
Diptera	Syrphidae	*Syritta orientalis* Macquart, 1842	PO118	43	2
Diptera	Syrphidae	*Syrphus ribesii* (Linnaeus, 1758)	PO119	9	0
Diptera	Syrphidae	*Syrphus* sp.1	PO120	12	0
Diptera	Syrphidae	*Xanthandrus comtus* (Harris, 1780)	PO121	5	0
Diptera	Syrphidae	*Xylota* sp.	PO122	243	8
Diptera	Tachinidae	*Blepharipa carbonata* (Mesnil, 1970)	PO123	91	11
Diptera	Tachinidae	*Blepharipa latigena* (Mesnil, 1970)	PO124	81	14
Diptera	Tachinidae	*Dexia* sp.	PO125	40	1
Diptera	Tachinidae	*Eophyllophila* sp.	PO126	46	3
Diptera	Tachinidae	*Exorista* sp.	PO127	32	1
Diptera	Tachinidae	*Gymnosoma* sp.	PO128	80	8
Diptera	Tachinidae	Tachinidae sp.1	PO129	33	2
Diptera	Tachinidae	Tachinidae sp.2	PO130	9	1
Diptera	Tephritidae	Tephritidae sp.1	PO131	16	0
Diptera	Tephritidae	Tephritidae sp.2	PO132	8	0
Diptera	Ulidiidae	Ulidiidae sp.1	PO133	28	6
Hemiptera	Anthocoridae	*Orius similis* Zheng	PO134	53	0
Hemiptera	Anthocoridae	*Orius* sp.	PO135	7	0
Hemiptera	Coreidae	*Cletus* sp.	PO136	43	2
Hemiptera	Coreidae	*Homoeocerus striicornis* Scott, 1874	PO137	59	1
Hemiptera	Lygaeidae	*Eysarcoris montivagus* (Distant, 1902)	PO138	4	0
Hemiptera	Lygaeidae	*Geocoris pallidipennis* (Costa, 1843)	PO139	66	1
Hemiptera	Lygaeidae	Lygaeidae sp.	PO140	71	0
Hemiptera	Lygaeidae	*Nysius ericae* (Schilling, 1829)	PO141	54	0
Hemiptera	Lygaeidae	*Nysius* sp.	PO142	1651	74
Hemiptera	Lygaeidae	*Sadoletus* sp.	PO143	278	27
Hemiptera	Miridae	*Apolygus* sp.1	PO144	28	0
Hemiptera	Miridae	*Apolygus* sp.2	PO145	4	0
Hemiptera	Miridae	*Deraeocoris* sp.	PO146	75	2
Hemiptera	Neididae	*Metatropis* sp.	PO147	87	3
Hemiptera	Reduviidae	*Sphedanolestes gularis* Hsiao, 1979	PO148	89	3
Hymenoptera	Andrenidae	*Andrena camellia* Wu, 1977	PO149	20	2
Hymenoptera	Andrenidae	*Andrena chekiangensis* Wu, 1977	PO150	6	2
Hymenoptera	Andrenidae	*Andrena crassepunctata* Cockerell, 1931	PO151	1	0
Hymenoptera	Andrenidae	*Andrena knuthi chinensis* Wu, 1982	PO152	18	1
Hymenoptera	Andrenidae	*Andrena striata* Wu, 1977	PO153	18	0
Hymenoptera	Apidae	*Amegilla florea* (Smith, 1879)	PO154	950	43
Hymenoptera	Apidae	*Amegilla parhypate* Lieftinck, 1975	PO155	7	0
Hymenoptera	Apidae	*Amegilla* sp.	PO156	70	1
Hymenoptera	Apidae	*Apis cerana* Fabricius, 1793	PO157	10	1
Hymenoptera	Apidae	*Bombus atripes* Smith, 1852	PO158	52	0
Hymenoptera	Apidae	*Bombus eximius* Smith, 1852	PO159	51	1
Hymenoptera	Apidae	*Bombus flavescens* Smith, 1852	PO160	46	3
Hymenoptera	Apidae	*Bombus ignitus* Smith, 1869	PO161	86	8
Hymenoptera	Apidae	*Bombus imitator* Pittioni, 1949	PO162	26	3
Hymenoptera	Apidae	*Bombus kulingensis* Cockerell, 1917	PO163	290	20
Hymenoptera	Apidae	*Bombus trifasciatus* Smith, 1852	PO164	575	37
Hymenoptera	Apidae	*Ceratina flavipes* Smith, 1879	PO165	21	4
Hymenoptera	Apidae	*Ceratina* sp.1	PO166	10	0
Hymenoptera	Apidae	*Ceratina* sp.2	PO167	29	2
Hymenoptera	Apidae	*Ceratina* sp.3	PO168	3	0

Hymenoptera	Apidae	*Crocisa emarginata* Lepeletier, 1841	PO169	5	1
Hymenoptera	Apidae	*Eucera sociabilis* Smith, 1873	PO170	18	1
Hymenoptera	Apidae	*Psithyrus pieli* Maa, 1948	PO171	77	3
Hymenoptera	Apidae	*Psithyrus turneri* Richards, 1929	PO172	21	2
Hymenoptera	Apidae	*Tetralonia chinensis* Smith,1854	PO173	38	4
Hymenoptera	Apidae	*Tetralonia floralia* Smith,1854	PO174	7	2
Hymenoptera	Apidae	*Xylocopa appendiculata* Smith, 1852	PO175	1	0
Hymenoptera	Apidae	*Xylocopa nasalis* Westwood, 1838	PO176	3	0
Hymenoptera	Apidae	*Xylocopa rufipes* Smith, 1852	PO177	5	0
Hymenoptera	Apidae	*Xylocopa sinensis* Smith, 1854	PO178	30	0
Hymenoptera	Apidae	*Xylocopa tranquabarorum* Swederus, 1787	PO179	99	2
Hymenoptera	Argidae	*Arge* sp.1	PO180	74	1
Hymenoptera	Argidae	*Arge* sp.2	PO181	30	2
Hymenoptera	Braconidae	*Aleiodes* sp.1	PO182	82	3
Hymenoptera	Braconidae	*Aleiodes* sp.2	PO183	48	1
Hymenoptera	Braconidae	*Apanteles* sp.1	PO184	199	16
Hymenoptera	Braconidae	*Apanteles* sp.2	PO185	164	12
Hymenoptera	Braconidae	*Doryctes* sp.1	PO186	17	0
Hymenoptera	Braconidae	*Doryctes* sp.2	PO187	6	0
Hymenoptera	Braconidae	*Macrocentrus* sp.	PO188	30	1
Hymenoptera	Crabronidae	*Cerceris quadrifasciata* (Panzer, 1799)	PO189	24	2
Hymenoptera	Colletidae	*Colletes gigas* Cockerell, 1918	PO190	14	0
Hymenoptera	Chalcididae	*Brachymeria* sp.1	PO191	31	2
Hymenoptera	Chalcididae	*Brachymeria* sp.2	PO192	168	12
Hymenoptera	Chalcididae	*Brachymeria* sp.3	PO193	190	5
Hymenoptera	Chrysididae	Chrysis sp.	PO194	32	0
Hymenoptera	Colletidae	*Hylaeus* sp.1	PO195	25	3
Hymenoptera	Colletidae	*Hylaeus* sp.2	PO196	123	5
Hymenoptera	Eumenidae	*Ancistrocerus antoni* (Cameron, 1900)	PO197	21	1
Hymenoptera	Eumenidae	*Anterhynchium flavomarginatum* (Smith, 1852)	PO198	640	21
Hymenoptera	Eumenidae	*Anterhynchium* sp.	PO199	534	26
Hymenoptera	Eumenidae	*Discoelius* sp.	PO200	4	0
Hymenoptera	Eumenidae	*Orancistrocerus aterrimus* (de Saussure, 1852)	PO201	29	1
Hymenoptera	Eumenidae	*Orancistrocerus drewseni* (de Saussure, 1857)	PO202	10	0
Hymenoptera	Eumenidae	*Pseumenes* sp.	PO203	13	0
Hymenoptera	Eumenidae	*Stenodynerus frauenfeldi* (de Saussure, 1867)	PO204	15	0
Hymenoptera	Eumenidae	*Stenodynerus pappi* Giordani Soika, 1976	PO205	1	0
Hymenoptera	Formicidae	*Camponotus albosparsus* Bingham, 1903	PO206	31	1
Hymenoptera	Formicidae	*Camponotus vitiosus* Smith, 1874	PO207	31	0
Hymenoptera	Formicidae	*Crematogaster osakensis* Forel, 1900	PO208	11	1
Hymenoptera	Formicidae	*Crematogaster* sp.1	PO209	9	0
Hymenoptera	Formicidae	*Crematogaster* sp.2	PO210	22	0
Hymenoptera	Formicidae	*Iridmyrmex anceps* (Roger, 1863)	PO211	40	2
Hymenoptera	Formicidae	*Liometopum sinense* Wheeler, 1921	PO212	24	0
Hymenoptera	Formicidae	*Monomorium intrudens* Smith, 1874	PO213	2	0
Hymenoptera	Formicidae	*Monomorium pharaonis* (Linnaeus, 1758)	PO214	17	0
Hymenoptera	Formicidae	*Monomorium* sp.	PO215	11	0
Hymenoptera	Formicidae	*Ochetellus glaber* (Mayr, 1862)	PO216	32	4
Hymenoptera	Formicidae	*Paratrechina bourbonica* (Forel, 1886)	PO217	6	0
Hymenoptera	Formicidae	*Polyrhachis vicina* Roger, 1863	PO218	24	0
Hymenoptera	Formicidae	*Pristomyrmex pungens* Mayr, 1866	PO219	15	2
Hymenoptera	Halictidae	*Halictus aerarius* Smith, 1873	PO220	201	10
Hymenoptera	Halictidae	*Lasioglossum eidmanni* (Blüthgen, 1926)	PO221	100	4
Hymenoptera	Halictidae	*Lasioglossum* sp.1	PO222	88	2
Hymenoptera	Halictidae	*Lasioglossum* sp.2	PO223	15	4
Hymenoptera	Halictidae	*Lasioglossum* sp.3	PO224	68	6
Hymenoptera	Halictidae	*Lasioglossum* sp.4	PO225	7	0
Hymenoptera	Halictidae	*Lasioglossum subopacum* (Smith, 1853)	PO226	39	0
Hymenoptera	Halictidae	*Lasioglossum zonulum* (Smith, 1948)	PO227	5	0
Hymenoptera	Halictidae	*Nomia megasoma* Cockerell, 1912	PO228	4	0

Hymenoptera	Halictidae	*Nomia* sp.	PO229	4	0
Hymenoptera	Halictidae	*Nomia thoracica* (Smith, 1875)	PO230	4	0
Hymenoptera	Halictidae	*Rhopalomelissa* sp.	PO231	29	6
Hymenoptera	Halictidae	*Sphecodes grahami* Cockerell, 1922	PO232	6	0
Hymenoptera	Halictidae	*Sphecodes* sp.1	PO233	6	0
Hymenoptera	Ichneumonidae	*Enicospilus* sp.	PO234	13	3
Hymenoptera	Ichneumonidae	*Gotra* sp.	PO235	24	3
Hymenoptera	Leucospidae	*Leucospis* sp.1	PO236	25	1
Hymenoptera	Leucospidae	*Leucospis* sp.2	PO237	22	0
Hymenoptera	Megachilidae	*Bathanthidium sibiricum* (Eversmann, 1852)	PO238	103	0
Hymenoptera	Megachilidae	*Callomegachile faceta rufojugata* Cockerell, 1931	PO239	6	0
Hymenoptera	Megachilidae	*Megachile monticola* Smith, 1853	PO240	47	3
Hymenoptera	Megachilidae	*Megachile nipponica* Cockerell, 1914	PO241	310	44
Hymenoptera	Megachilidae	*Megachile remota* Smith, 1879	PO242	13	0
Hymenoptera	Megachilidae	*Megachile* sp.	PO243	65	3
Hymenoptera	Melittidae	*Macropis immaculata* (Wu, 1965)	PO244	87	7
Hymenoptera	Polistidae	*Polistes jokahamae* Radoszkowski, 1887	PO245	26	4
Hymenoptera	Pompilidae	*Batozonellus* sp.	PO246	157	12
Hymenoptera	Pompilidae	Pompilidae sp.1	PO247	50	1
Hymenoptera	Pompilidae	Pompilidae sp.2	PO248	48	5
Hymenoptera	Scoliidae	*Campsomeris annulata* (Fabricius, 1793)	PO249	35	2
Hymenoptera	Scoliidae	*Campsomeris prismatica* (Smith, 1855)	PO250	18	2
Hymenoptera	Scoliidae	*Scolia formosicola* Betrem, 1928	PO251	37	3
Hymenoptera	Scoliidae	*Scolia nobilitata* Fabricius, 1805	PO252	9	1
Hymenoptera	Scoliidae	*Scolia oculata* (Matsumura, 1911)	PO253	2	0
Hymenoptera	Scoliidae	*Scolia sinensis* Saussure, 1846	PO254	38	0
Hymenoptera	Scoliidae	*Scolia* sp.	PO255	18	2
Hymenoptera	Scoliidae	*Triscolia ardens* (Smith 1855)	PO256	16	0
Hymenoptera	Sphecidae	*Ammophila atripes* F.Smith, 1852	PO257	2	0
Hymenoptera	Sphecidae	*Cerceris* sp.	PO258	2	0
Hymenoptera	Sphecidae	*Chalybion japonicum* (Gribodo, 1883)	PO259	21	0
Hymenoptera	Sphecidae	*Ectemnius* sp.	PO260	3	0
Hymenoptera	Tenthredinidae	*Athalia* sp.	PO261	4	1
Hymenoptera	Tenthredinidae	*Mesonura rufonota* Rohwer	PO262	27	1
Hymenoptera	Tenthredinidae	*Tenthredo* sp.	PO263	20	1
Hymenoptera	Vespidae	*Vespa velutina* Lepeletier, 1836	PO264	15	0
Lepidoptera	Ctenuchidae	*Amata germana* (Felder, 1862)	PO265	45	1
Lepidoptera	Ctenuchidae	*Amata* sp.	PO266	48	1
Lepidoptera	Geometridae	*Calospilos suspecta* (Warren, 1894)	PO267	20	0
Lepidoptera	Hesperiidae	*Aeromachus inachus* Menetries, 1859	PO268	12	1
Lepidoptera	Hesperiidae	*Ampittia virgata* Leech, 1890	PO269	23	0
Lepidoptera	Hesperiidae	*Celaenorrhinus maculosus* (C. & R. Felder, 1867)	PO270	14	0
Lepidoptera	Hesperiidae	*Halpe porus* (Mabille, 1877)	PO271	43	1
Lepidoptera	Hesperiidae	Hesperiidae sp.	PO272	24	1
Lepidoptera	Hesperiidae	*Parnara guttata* (Bremer & Grey, 1853)	PO273	918	33
Lepidoptera	Hesperiidae	*Polytremis* sp.	PO274	57	2
Lepidoptera	Lycaenidae	*Acytolepis puspa* (Horsfield, 1828)	PO275	31	1
Lepidoptera	Lycaenidae	*Celastrina argiolus* (Linnaeus, 1758)	PO276	7	0
Lepidoptera	Lycaenidae	*Curetis acuta* Moore, 1877	PO277	32	0
Lepidoptera	Lycaenidae	*Everes argiades* (Pallas, 1771)	PO278	17	0
Lepidoptera	Lycaenidae	*Pseudozizeeria maha* (Kollar, [1844])	PO279	58	2
Lepidoptera	Lycaenidae	*Tongeia filicaudis* (Pryer, 1877)	PO280	17	0
Lepidoptera	Lycaenidae	*Udara albocaerulea* (Moore, 1879)	PO281	21	2
Lepidoptera	Nymphalidae	*Argyreus hyperbius* (Linnaeus, 1763)	PO282	38	0
Lepidoptera	Nymphalidae	*Athyma zeroca* Moore, 1872	PO283	9	1
Lepidoptera	Nymphalidae	*Damora sagana* (Doubleday, 1847)	PO284	7	2
Lepidoptera	Nymphalidae	*Kaniska canace* (Linnaeus, 1763)	PO285	7	1
Lepidoptera	Nymphalidae	*Limenitis helmanni* Lederer, 1853	PO286	7	0
Lepidoptera	Nymphalidae	*Limenitis sulpitia* (Cramer, [1779])	PO287	7	0
Lepidoptera	Nymphalidae	*Neptis clinia* Moore, 1872	PO288	2	0

Lepidoptera	Nymphalidae	*Neptis sappho* (Pallas, 1771)	PO289	14	1
Lepidoptera	Nymphalidae	*Neptis* sp.	PO290	25	1
Lepidoptera	Nymphalidae	*Polyura narcaea* Hewitson, 1854	PO291	3	0
Lepidoptera	Nymphalidae	*Vanessa cardui* (Linnaeus, 1758)	PO292	25	0
Lepidoptera	Papilionidae	*Achillides bianor* Cramer, [1777]	PO293	22	2
Lepidoptera	Papilionidae	*Byasa alcinous* (Klug, 1836)	PO294	24	0
Lepidoptera	Papilionidae	*Graphium sarpedon* (Linnaeus, 1758)	PO295	7	0
Lepidoptera	Papilionidae	*Papilio machaon* Linnaeus, 1758	PO296	43	0
Lepidoptera	Papilionidae	*Papilio protenor* Cramer, 1775	PO297	66	1
Lepidoptera	Pieridae	*Colias erate* (Esper, 1805)	PO298	103	3
Lepidoptera	Pieridae	*Eurema blanda* (Boisduval, 1836)	PO299	146	5
Lepidoptera	Pieridae	*Eurema hecabe* (Linnaeus, 1758)	PO300	4	0
Lepidoptera	Pieridae	*Pieris rapae* (Linnaeus, 1758)	PO301	5	0
Lepidoptera	Pyralidae	*Bocchoris inspersalis* Zeller, 1852	PO302	7	0
Lepidoptera	Riodinidae	*Stiboges nymphidia* Butler, 1876	PO303	6	0
Lepidoptera	Satyridae	*Mycalesis gotama* Moore, 1857	PO304	50	0
Lepidoptera	Satyridae	*Neope muirheadi* (C. & R. Felder, 1862)	PO305	20	1
Lepidoptera	Satyridae	*Ypthima argus* (Butler, 1878)	PO306	10	0
Lepidoptera	Satyridae	*Ypthima motschulskyi* Bremer & Grey, 1853	PO307	2	0
Lepidoptera	Sphingidae	*Cephonodes hylas* (Linnaeus, 1771)	PO308	169	4
Lepidoptera	Sphingidae	*Macroglossum corythus* Walker, 1856	PO309	8	1
Lepidoptera	Sphingidae	*Sataspes infernalis* (Westwood, 1847)	PO310	50	0
Lepidoptera	Zygaenidae	*Histia rhodope* (Cramer, [1775])	PO311	12	0
Lepidoptera	Zygaenidae	*Illiberis Pruni* Dyar, 1905	PO312	3	0
Lepidoptera	Zygaenidae	*Pidorus atratus* Butler, 1877	PO313	20	4

Table S4. Summary of partial regression coefficients for the final piecewise SEM model describing the relationships among island attributes, community structure and network architecture.

Response	Predictor	Parameter estimate	Standard error	df	t-value	Standard estimate
Floral resources	Decreased area	-0.543	0.042	39	164.488	**-0.582*****
	Proximity to edge	2.651	0.099	40	717.649	**0.741*****
Plant richness	Decreased area	-0.193	0.016	82	-11.801	**-0.485*****
	Proximity to edge	1.120	0.079	82	14.171	**0.734*****
Pollinator richness	Pollinator abundance	0.612	0.013	82	48.650	**0.871*****
Pollinator abundance	Decreased area	-0.267	0.051	82	-5.237	**-0.157*****
	Proximity to edge	2.245	0.201	82	11.195	**0.114*****
	Decreased area × Proximity to edge	0.049	0.013	82	3.608	**0.018*****
	Floral resources	0.305	0.073	82	4.170	**0.053*****
Relative connectance	Decreased area	0.007	0.009	64	0.621	-0.066
	Proximity to edge	-0.071	0.048	71	2.090	-0.045
	Decreased area × Proximity to edge	-0.035	0.009	54	15.417	**-0.180*****
	Plant richness	-0.210	0.028	72	53.812	**-0.342*****
	Pollinator richness	-0.197	0.047	75	16.860	**-0.192*****
	Pollinator abundance	0.168	0.036	74	20.174	**0.210*****
Nestedness	Distance to mainland	1.557	0.681	38	5.237	**0.117***
	Pollinator richness	-11.838	2.156	76	28.793	**-0.282*****
	Pollinator abundance	12.009	1.515	74	60.185	**0.407*****
Modularity	Plant richness	0.099	0.034	77	7.919	**0.174****
	Pollinator richness	0.144	0.051	77	7.629	**0.170****
	Pollinator abundance	-0.196	0.035	77	29.808	**-0.334*****
	Relative connectance	-0.616	0.106	77	32.253	**-0.350*****
Robustness	Nestedness	0.013	0.001	68	84.030	**0.725*****
	Modularity	0.405	0.088	79	20.157	**0.356*****
~~Floral resources	~~Plant richness	0.388	-	82	3.747	**0.388*****
~~Modularity	~~NODF	-0.275	-	82	-2.545	**-0.275****

Statistical significance: * $P < 0.05$; ** $P < 0.01$; *** $P < 0.001$. Bold signifies $P < 0.05$; ~~ Correlated predictors.

Table S5. Summary of direct, indirect, and total effects for each response variable.

Response variables	Variables	Total effect (95% CI)	Direct effect (95% CI)	Indirect effect (95% CI)
Floral resources	Decreased area	-0.582 [-0.726, -0.459] *	-0.582 [-0.726, -0.459] *	-
	Proximity to edge	0.741 [0.630, 0.805] *	0.741 [0.630, 0.805] *	-
Plant richness	Decreased area	-0.485 [-0.612, -0.345] *	-0.485 [-0.612, -0.345] *	-
	Proximity to edge	0.734 [0.621, 0.810] *	0.734 [0.621, 0.810] *	-
Pollinator richness	Decreased area	-0.163 [-0.260, -0.100] *	-	-0.163 [-0.260, -0.100] *
	Proximity to edge	0.134 [0.090, 0.191] *	-	0.134 [0.090, 0.191] *
	Decreased area × Proximity to edge	0.016 [0.006, 0.039] *	-	0.016 [0.006, 0.039] *
	Floral resources	0.046 [0.012, 0.093] *	-	0.046 [0.012, 0.093] *
	Pollinator abundance	0.871 [0.794, 0.922] *	0.871 [0.794, 0.922] *	-
Pollinator abundance	Decreased area	-0.188 [-0.319, -0.114] *	-0.157 [-0.299, -0.065] *	-0.031 [-0.054, -0.013] *
	Proximity to edge	0.153 [0.112, 0.211] *	0.114 [0.082, 0.155] *	0.039 [0.009, 0.080] *
	Decreased area × Proximity to edge	0.018 [0.007, 0.043] *	0.018 [0.007, 0.043] *	-
	Floral resources	0.053 [0.015, 0.102] *	0.053 [0.015, 0.102] *	-
Relative connectance	Decreased area	0.158 [0.124, 0.223] *	-	0.158 [0.124, 0.223] *
	Proximity to edge	-0.245 [-0.322, -0.199] *	-	-0.245 [-0.322, -0.199] *
	Decreased area × Proximity to edge	-0.185 [-0.244, -0.154] *	-0.185 [-0.244, -0.155] *	0.001 [0.000, 0.003]
	Floral resources	0.002 [0.001, 0.006] *	-	0.002 [0.001, 0.006] *
	Plant richness	-0.344 [-0.443, -0.286] *	-0.344 [-0.443, -0.286] *	-
	Pollinator richness	-0.175 [-0.292, -0.089] *	-0.175 [-0.292, -0.089] *	-
	Pollinator abundance	0.047 [0.028, 0.087] *	0.199 [0.140, 0.306] *	-0.152 [-0.261, -0.072] *
Nestedness	Decreased area	-0.030 [-0.066, -0.017] *	-	-0.030 [-0.066, -0.017] *
	Proximity to edge	0.025 [0.023, 0.029] *	-	0.025 [0.023, 0.029] *
	Decreased area × Proximity to edge	0.003 [0.002, 0.006] *	-	0.003 [0.002, 0.006] *
	Distance to mainland	0.117 [0.044, 0.198] *	0.117 [0.044, 0.198] *	-
	Floral resources	0.008 [0.004, 0.016] *	-	0.008 [0.004, 0.016] *
	Pollinator richness	-0.282 [-0.393, -0.223] *	-0.282 [-0.393, -0.223] *	-
	Pollinator abundance	0.161 [0.133, 0.227] *	0.407 [0.347, 0.531] *	-0.246 [-0.331, -0.193] *
Modularity	Decreased area	-0.105 [-0.192, -0.068] *	-	-0.105 [-0.192, -0.068] *
	Proximity to edge	0.185 [0.139, 0.289] *	-	0.185 [0.139, 0.289] *
	Decreased area × Proximity to edge	0.061 [0.030, 0.127] *	-	0.061 [0.030, 0.127] *
	Floral resources	-0.011 [-0.019, -0.006] *	-	-0.011 [-0.019, -0.006] *
	Plant richness	0.294 [0.229, 0.429] *	0.174 [0.073, 0.308] *	0.120 [0.051, 0.224] *
	Pollinator richness	0.231 [0.100, 0.393] *	0.170 [0.028, 0.349] *	0.061 [0.035, 0.116] *
	Pollinator abundance	-0.203 [-0.251, -0.178] *	-0.334 [-0.530, -0.189] *	0.132 [0.001, 0.303] *
	Relative connectance	-0.350 [-0.516, -0.15] *	-0.350 [-0.516, -0.150] *	-
Robustness	Decreased area	-0.059 [-0.130, -0.023] *	-	-0.059 [-0.130, -0.023] *
	Proximity to edge	0.083 [0.027, 0.158] *	-	0.083 [0.027, 0.158] *
	Decreased area × Proximity to edge	0.024 [0.003, 0.073] *	-	0.024 [0.003, 0.073] *
	Distance to mainland	0.085 [0.043, 0.151] *	-	0.085 [0.043, 0.151] *
	Floral resources	0.002 [0.001, 0.005] *	-	0.002 [0.001, 0.005] *
	Plant richness	0.105 [0.022, 0.228] *	-	0.105 [0.022, 0.228] *
	Pollinator richness	-0.122 [-0.250, -0.064] *	-	-0.122 [-0.250, -0.064] *
	Pollinator abundance	0.045 [0.002, 0.086] *	-	0.045 [0.002, 0.086] *
	Relative connectance	-0.125 [-0.303, -0.005] *	-	-0.125 [-0.303, -0.005] *
	Nestedness	0.725 [0.516, 0.850] *	0.725 [0.516, 0.850] *	-
	Modularity	0.356 [0.027, 0.602] *	0.356 [0.027, 0.602] *	-

The “*” represents the effects that are significant, i.e., confidence intervals (CI) do not overlap zero.
