# Supplementary material for: Forest edges increase pollinator network robustness to extinction with declining area
Source: Nat Ecol Evol. 2023 Jan 30;7(3):393–404. doi: 10.1038/s41559-022-01973-y (PMC9998274; doi:10.1038/s41559-022-01973-y)
Supplement: Supplementary file 2 — Reporting Summary [file 41559_2022_1973_MOESM2_ESM.pdf]

nature portfolio

Corresponding author(s): Ping Ding

Last updated by author(s): Oct 31, 2022

Reporting Summary

Nature Portfolio wishes to improve the reproducibility of the work that we publish. This form provides structure for consistency and transparency in reporting. For further information on Nature Portfolio policies, see our Editorial Policies and the Editorial Policy Checklist.

Statistics

For all statistical analyses, confirm that the following items are present in the figure legend, table legend, main text, or Methods section.

n/a	Confirmed	
☐	☒	The exact sample size (*n*) for each experimental group/condition, given as a discrete number and unit of measurement
☐	☒	A statement on whether measurements were taken from distinct samples or whether the same sample was measured repeatedly
☐	☒	The statistical test(s) used AND whether they are one- or two-sided<br>*Only common tests should be described solely by name; describe more complex techniques in the Methods section.*
☐	☒	A description of all covariates tested
☐	☒	A description of any assumptions or corrections, such as tests of normality and adjustment for multiple comparisons
☐	☒	A full description of the statistical parameters including central tendency (e.g. means) or other basic estimates (e.g. regression coefficient) AND variation (e.g. standard deviation) or associated estimates of uncertainty (e.g. confidence intervals)
☐	☒	For null hypothesis testing, the test statistic (e.g. *F*, *t*, *r*) with confidence intervals, effect sizes, degrees of freedom and *P* value noted<br>*Give P values as exact values whenever suitable.*
☒	☐	For Bayesian analysis, information on the choice of priors and Markov chain Monte Carlo settings
☐	☒	For hierarchical and complex designs, identification of the appropriate level for tests and full reporting of outcomes
☐	☒	Estimates of effect sizes (e.g. Cohen's *d*, Pearson's *r*), indicating how they were calculated

Our web collection on statistics for biologists contains articles on many of the points above.

Software and code

Policy information about availability of computer code

Data collection	No software was used for data collection.
Data analysis	All analyses were performed with the open source R (version 4.0.1). We used the bipartite (version 2.16) and vegan (version 2.5-7) packages for plant-pollinator network architecture analysis and the piecewiseSEM package (version 2.1.0) for piecewise structural equation models. Direct, indirect, and total effects from piecewiseSEM were calculated using the semEff package (version 0.6.0). Null models for plant-pollinator community structure and network architecture were generated using our own code written in R. Plots were generated with R (version 4.0.1) and ggplot2 (version 3.3.5) or in LaTeX (version 2.9.2).

For manuscripts utilizing custom algorithms or software that are central to the research but not yet described in published literature, software must be made available to editors and reviewers. We strongly encourage code deposition in a community repository (e.g. GitHub). See the Nature Portfolio guidelines for submitting code & software for further information.

Data

Policy information about availability of data

All manuscripts must include a data availability statement. This statement should provide the following information, where applicable:

- Accession codes, unique identifiers, or web links for publicly available datasets
- A description of any restrictions on data availability
- For clinical datasets or third party data, please ensure that the statement adheres to our policy

Scripts for null model generation are available in figshare at https://doi.org/10.6084/m9.figshare.20477889
All raw data and processed code will be made available upon reasonable request.

Human research participants

Policy information about studies involving human research participants and Sex and Gender in Research.

Reporting on sex and gender	This study did not involve human participants.
Population characteristics	This study did not involve human participants.
Recruitment	This study did not involve human participants.
Ethics oversight	This study did not involve human participants.

Note that full information on the approval of the study protocol must also be provided in the manuscript.

Field-specific reporting

Please select the one below that is the best fit for your research. If you are not sure, read the appropriate sections before making your selection.

☐ Life sciences ☐ Behavioural & social sciences ☒ Ecological, evolutionary & environmental sciences

For a reference copy of the document with all sections, see nature.com/documents/nr-reporting-summary-flat.pdf

Ecological, evolutionary & environmental sciences study design

All studies must disclose on these points even when the disclosure is negative.

Study description	We conducted surveys of plant-pollinator interactions at paired edge versus interior sites on 41 islands and 16 mainland sites over 3 years. Mainland sites were selected to achieve maximum spatial coverage around the lake margin, and similarity of vegetation types to those on the sampled islands. We established paired transect lines (100 × 4 m), with one along the edge and one extending perpendicular from the edge into forest interior at each site. On islands, the number of pairs of transects varied from 1 to 16, and was roughly proportional to (ln-transformed) island size. On islands with more than two pairs of transects, each pair was separated by ≥ 0.5 km.
Research sample	Our research samples consisted of plant and pollinators species, as well as their interactions.
Sampling strategy	Along each transect, we observed individual flowering branches of shrubs and trees, or the whole plant in the case of herbaceous plants under 3.5 m from the ground. We walked each edge transect at a mean pace of 7m / min and each interior transect at mean pace of 10m / min (due to the larger number of flowering plants at edges we used a 15-minute survey interval for edge transects, but a 10-minute survey interval for interior transects).
Data collection	Observations were carried out only in calm and sunny weather, from 8:30 AM to 12:00 noon and from 1:00 PM to 5:00 PM. We considered an insect to be a putative pollinator only if it was touching the anthers and/or stigmas of the flowers.
Timing and spatial scale	We observed transects at multiple times throughout the season on 41 islands and 16 mainland sites over 3 years. We sampled once every two weeks, on average, with six surveys conducted at each site from 20th April to 20th July in 2017, seven surveys at each site from 23rd March to 14th July in 2018 and seven surveys at each site from 13th March to 20th July in 2019 (i.e., 20 surveys in total at each site).
Data exclusions	No data were excluded from the analysis.
Reproducibility	All data necessary to repeat the analyses will be made publicly available.
Randomization	Transect selection was stratified across dominant spatial and temporal gradients of variation, in order to standardise sample collection.

Null model analysis used a fully-random and stratified-random procedures to draw null samples from the mainland reference pool of plant-pollinator interactions.

Blinding	Not applicable to this study.

Did the study involve field work? ☒ Yes ☐ No

Field work, collection and transport

Field conditions	The main habitat type is unmanaged secondary forest (typical coverage ~90%) and the dominant plant species is Pinus massoniana. The climate is typical of the subtropical monsoon zone and is highly seasonal. Median annual precipitation in this area is 1430 mm, mainly concentrated in the rainy season between April and June. The average annual temperature is 17.0°C, ranging from -7.6 °C to 41.8 °C.
Location	The study was carried out in the Thousand Island Lake, Zhejiang Province, eastern China (29°22"–29°50" N, 118°34"–119°15" E). This large artificial reservoir was created in 1959 by the construction of the Xin'anjiang Dam for hydroelectricity.
Access & import/export	The research was carried out under the Xin'an River Ecological Development Group Corporation and Forestry Bureau of Chun'an County permit.
Disturbance	No disturbance was caused in the study sites.

Reporting for specific materials, systems and methods

We require information from authors about some types of materials, experimental systems and methods used in many studies. Here, indicate whether each material, system or method listed is relevant to your study. If you are not sure if a list item applies to your research, read the appropriate section before selecting a response.

Materials & experimental systems

n/a	Involved in the study
☒	☐ Antibodies
☒	☐ Eukaryotic cell lines
☒	☐ Palaeontology and archaeology
☒	☐ Animals and other organisms
☒	☐ Clinical data
☒	☐ Dual use research of concern

Methods

n/a	Involved in the study
☒	☐ ChIP-seq
☒	☐ Flow cytometry
☒	☐ MRI-based neuroimaging
